# Study of the Microbiome of the Cretan Sour Cream Staka Using Amplicon Sequencing and Shotgun Metagenomics and Isolation of Novel Strains with an Important Antimicrobial Potential

**DOI:** 10.3390/foods13071129

**Published:** 2024-04-08

**Authors:** Konstantinos Papadimitriou, Marina Georgalaki, Rania Anastasiou, Athanasia-Maria Alexandropoulou, Eugenia Manolopoulou, Georgia Zoumpopoulou, Effie Tsakalidou

**Affiliations:** 1 Laboratory of Food Quality Control and Hygiene, Department of Food Science and Human Nutrition, Agricultural University of Athens, Iera Odos 75, 11855 Athens, Greece; 2 Laboratory of Dairy Research, Department of Food Science and Human Nutrition, Agricultural University of Athens, Iera Odos 75, 11855 Athens, Greece; mgeor@aua.gr (M.G.); ranastasiou@aua.gr (R.A.); siaal22@yahoo.gr (A.-M.A.); mae@aua.gr (E.M.); gz@aua.gr (G.Z.); et@aua.gr (E.T.)

**Keywords:** artisanal, microbial ecosystem, starter culture, bacteriocin, foodborne, oral, medical, pathogen, dead cells, VBNC

## Abstract

Staka is a traditional Greek sour cream made mostly from spontaneously fermented sheep milk or a mixture of sheep and goat milk. At the industrial scale, cream separators and starter cultures may also be used. Staka is sometimes cooked with flour to absorb most of the fat. In this study, we employed culture-based techniques, amplicon sequencing, and shotgun metagenomics to analyze the Staka microbiome for the first time. The samples were dominated by *Lactococcus* or *Leuconostoc* spp. Most other bacteria were lactic acid bacteria (LAB) from the *Streptococcus* and *Enterococcus* genera or Gram-negative bacteria from the *Buttiauxella*, *Pseudomonas*, *Enterobacter*, *Escherichia*-*Shigella*, and *Hafnia* genera. *Debaryomyces*, *Kluyveromyces*, or *Alternaria* were the most prevalent genera in the samples, followed by other yeasts and molds like *Saccharomyces*, *Penicillium*, *Aspergillus*, *Stemphylium*, *Coniospotium*, or *Cladosporium* spp. Shotgun metagenomics allowed the species-level identification of *Lactococcus lactis*, *Lactococcus raffinolactis*, *Streptococcus thermophilus*, *Streptococcus gallolyticus*, *Escherichia coli*, *Hafnia alvei*, *Streptococcus parauberis*, and *Enterococcus durans*. Binning of assembled shotgun reads followed by recruitment plot analysis of single reads could determine near-complete metagenome assembled genomes (MAGs). Culture-dependent and culture-independent analyses were in overall agreement with some distinct differences. For example, lactococci could not be isolated, presumably because they had entered a viable but not culturable (VBNC) state or because they were dead. Finally, several LAB, *Hafnia paralvei*, and *Pseudomonas* spp. isolates exhibited antimicrobial activities against oral or other pathogenic streptococci, and certain spoilage and pathogenic bacteria establishing their potential role in food bio-protection or new biomedical applications. Our study may pave the way for additional studies concerning artisanal sour creams to better understand the factors affecting their production and the quality.

## 1. Introduction

Dairy products with high lipid content can have added nutritious value as milk lipids represent a good dietary source of essential fatty acids, such as linoleic and α-linolenic acid, as well as fat-soluble vitamins, such as retinol, α-tocopherol and β-carotene [1]. Bioactive fatty acids, such as butyric, oleic, and conjugated linoleic acid (CLA), may also play key roles in the prevention of certain diseases [2,3,4]. Interestingly, although dairy food consumption has been positively correlated with cardiovascular risk in the past, recent observations suggest a potential inverse association of fermented dairy food consumption with cardiovascular problems and type 2 diabetes [5]. Additionally, the fatty acid composition of milk affects the physical properties, organoleptic quality, and oxidative stability of dairy products [6,7].

Fermented milk products of high fat content encompass fermented sour cream and acidified sour cream (18–20% fat), as well as fermented butter (80–90% fat). Fermented cream, also known as ripened cream [8,9], is manufactured from standardized, homogenized, and heat-treated sweet cream after fermentation with lactic acid bacteria (LAB). It is a relatively heavy, viscous product with a delicate, lactic acid taste and a balanced, pleasant, buttery-like aroma associated with the desirable flavor-active compounds of diacetyl, acetoin, δ-decalactone, and 2-methyl-3-furanthiol [10,11]. Additionally, acidified sour cream is prepared with safe and suitable acidifiers, such as lactic acid and citric acid, with or without the use of LAB [12,13]. Finally, fermented butter is made after churning fermented/cultured cream [13].

At the industrial level, mixed strains of mesophilic LAB are being used as starter cultures for sour cream production, mainly belonging to *Lactococcus lactis* subsp. *lactis*, *Lactococcus lactis* subsp. *cremoris*, *Lactococcus lactis* subsp. *lactis* biovar *diacetylactis*, *Leuconostoc mesenteroides* subsp. *cremoris,* and *Leuconostoc citrovorum* [10].These species are used as aroma producers, converting citrate into diacetyl, which is one of the major flavor compounds responsible for the typical sour cream flavor [14]. In many countries, though, artisanal sour cream is produced by keeping cream at a suitable temperature to allow autochthonous LAB to perform spontaneous fermentation [15]. A plethora of traditionally fermented cream and butter types are produced worldwide, such as Crème Fraiche in France, Kaymak in Turkey, Crema Espesa in Mexico, Pomazánkové Máslo in Czech Republic, Jiaoke in Mongolia, China, traditional sour cream in Ukraine, Suero Costeño in Colombia, and smetana/śmietana/śmietanka mainly in Russia and Poland [16,17,18,19].

Nowadays, amplicon sequencing and shotgun metagenomics are the main tools used to study the microbiome of artisanal dairy products in depth [20,21]. Amplicon sequencing refers to the sequencing of targeted amplified loci like the 16S rDNA of bacteria or the internal transcribed spacer (ITS) of fungi found in the metagenomic DNA of food samples and it usually resolves the microbiome composition at the genus level. In contrast, shotgun metagenomics relies on the sequencing of the entire metagenomic DNA fraction and can provide species- or even strain-level information of the microbiome under investigation along with in silico predictions of its functional properties. These approaches allow the rapid and comprehensive analysis of the microbial communities in dairy products and can reveal important aspects of their technology, quality, and safety. The microbiology of sour cream products has been reviewed in the recent past to an extent [22]. So far, very few such studies exist, involving only amplicon sequencing approaches. Two studies concern the characterization of the microbiome of traditional sour cream/butter products produced in Russia [23,24], and one concerns the analysis of the microbiome of the traditional “Suero Costeño” sour cream produced in Colombia [19].

Staka, also called “Anthogalo” (milk flower), is a traditional Greek sour cream, which has risen at the surface of the spontaneously fermented sheep or mixtures of sheep and goat milk [25]. The cream is naturally fermented in traditional clay pots called “kounenoi” and/or exposed to air and sun depending on the region and season of the year (personal communication). It can also be produced using separators with the addition of starter cultures. Sometimes, Staka is cooked with flour, and in this case, most of the butter is separated and called “Stakovoutyro” (Staka butter), while the cooked product is also called Staka. Because of its rich taste and creamy mouthfeel, it has several culinary uses, and the “stakopilafo” or “gamopilafo”, the special rice dish prepared in weddings, is the most known and appreciated one.

Recently, the microbiota of one Cretan Staka sample was investigated using a combination of culture-dependent methods and MALDI-TOF for the identification of the microbial isolates [25]. Following up these preliminary results, the aim of the current study was to analyze the microbial ecosystem of Staka in depth using a combination of culture-based microbiological analysis, amplicon sequencing, and shotgun metagenomics.

## 2. Materials and Methods

### 2.1. Samples

Five Staka samples, deriving from two geographical regions of Crete, namely Chania (Western Crete; Staka 1, 2, 3) and Sitia (Eastern Crete; Staka 4, 5), were included in the present study (Table 1). Four samples (Staka 1, 2, 3, 5) were commercial and purchased from mini markets in Athens or Chania; all were produced from pasteurized milk, while Staka 5 was cooked with wholemeal flour. Staka 4 was homemade, produced from raw milk, and cooked with wheat flour. One sample was analyzed for each Staka. For each Staka sample, three technical replicates were performed in the physicochemical and microbiological analyses.

### 2.2. Physicochemical Analyses

The pH of the Staka samples was measured using a pH meter (827 pH lab, Metrohm Herisau, Switzerland). Titratable acidity was measured by titrating 10 g sample with a standard solution of 0.1 Ν sodium hydroxide and phenolphthalein (Merck Darmstadt, Germany) as pH indicator and expressed as % lactic acid (*w*/*w*). The moisture content was measured according to ISO method [26]; ash content was determined according to [27]; and fat content was determined using the Gerber-van Gulik method [28]. The Kjeldahl method was used for the determination of total nitrogen (TN), which was expressed as protein content (% *w*/*w*) [28]. Finally, lactose, organic acids, and ethanol were determined by HPLC analysis [29].

### 2.3. Microbiological Analysis

The following groups of microorganisms were enumerated: (1) total mesophilic bacteria on plate count agar (PCA, Biokar Diagnostics, Beauvais, France) at 30 °C for 3 days; (2) psychrotrophic bacteria on PCA at 7 °C for 10 days; (3) lipolytic bacteria on PCA containing 1% *v*/*v* tributyrin (Sigma Aldrich Co., St. Louis, MO, USA) at 30 °C for 3 days; (4) proteolytic bacteria on PCA containing 10% *w*/*v* reconstituted skim milk (Oxoid, Hampshire, UK) at 30 °C for 48 h; (5) thermophilic LAB on MRS agar adjusted at pH 5.4 (presumably lactobacilli) (Biokar Diagnostics) at 42 °C for 48 h anaerobically (double agar layer); (6) mesophilic LAB in MRS agar adjusted at pH 5.4 (presumably lactobacilli) at 22 °C for 3 days anaerobically (double agar layer); (7) thermophilic LAB on M17 agar (presumably cocci) (Biokar Diagnostics) at 42 °C for 48 h; (8) mesophilic LAB on M17 agar (presumably cocci) at 22 °C for 48 h; (9) non-starter lactic acid bacteria (NSLAB) on Rogosa agar (Biokar Diagnostics) at 30 °C for 5 days, anaerobically (double agar layer); (10) enterococci on KAA agar (Merck) at 37 °C for 24 h; (11) coliforms on VRBL agar (Biokar Diagnostics) at 37 °C for 24 h; (12) *Pseudomonas* spp. on Pseudomonas agar base (Biokar Diagnostics), at 30 °C for 48 h; (13) yeasts and molds on YGC agar (Merck) at 25 °C for 3–4 days. Based on morphology (shape, color, and size), colonies were collected from MRS (42 and 22 °C), M17 (42 and 22 °C), Rogosa, KAA, PCA, PCA-tributyrin, and PCA-milk purified by repetitive streaking and stored at −80 °C in nutrient broth containing 20% *v*/*v* glycerol for further study.

### 2.4. Amplicon and Shotgun Metagenomics Sequencing and Analysis

Metagenomic DNA was isolated by the protocol described previously [30]. All the procedures for the DNA amplification, the construction of the different libraries, the Illumina sequencing, and preliminary quality control have been described before [30,31]. Sequencing was performed at Molecular Research DNA (MR DNA, Shallowater, TX, USA). Amplicon sequences and/or shotgun metagenomics were analyzed as described before with the CLC genomics workbench 11.0.1 (Qiagen, Hilden, Germany), the BusyBee web tool [32], and the metagenomics rapid annotation was analyzed using subsystems technology (MG-RAST) server version 4.0.3 [33]. During analysis, reads deriving from *Cellulosimicrobium* sp. Were removed from all sequence datasets due to the contamination by the lyticase used for the lysis of yeasts [34,35]. Finally, shotgun sequencing reads were aligned against different reference genomes in the CLC genomics workbench 11.0.1 and were processed into recruitment plots with the Recplot_4 R package (https://github.com/KGerhardt/Recplot_4, accessed on 1 February 2024) using default parameters. Reference genomes were selected after manually determining the best BLASTn hits of at least four random contigs from each of the top four species in abundance according to results of the BusyBee web tool (i.e., *Lc. lactis*, *Lactococcus raffinolactis*, *Streptococcus thermophilus,* and *Streptococcus gallolyticus*).

### 2.5. Typing and Identification of Isolates with Rep-PCR Fingerprinting and 16S rDNA Sequencing

A previously reported protocol was employed for bacterial DNA extraction using 2 mL of fresh overnight cultures in the exponential phase and fingerprinting of the new isolates was performed by repetitive element palindromic PCR (rep-PCR) [36]. A SimpliAmp™ Thermal Cycler (ThermoFisher Scientific, Sunnyvale, CA, USA) was used for the PCR. Rep-PCR fingerprint clustering was performed by BioNumerics v. 6.0 (Applied Maths, Ghent, Belgium).

Identification at the species level of bacterial isolates was performed by 16S rDNA sequencing [37]. The NucleoSpin^®^ Gel and PCR Clean-up (Macherey-Nagel, Duren, Germany) were used for DNA purification after electrophoresis. BLASTn was used for the identification at the species level.

### 2.6. Species Discrimination by Biochemical Tests

To discriminate species with ambiguous 16S rDNA sequencing results, several isolates were subjected to biochemical tests. Isolates were first grown as follows: *Enterococcus* spp. in M17 broth at 37 °C, *Lacticaseibacillus* spp. and *Leuconostoc* spp. in MRS broth at 30 °C, and *Hafnia* spp. in nutrient broth at 30 °C. Discrimination of enterococcal species was performed using mannitol salt agar (Lab M, Heywood, Lancashire, UK), taking into consideration the ability of *E. faecalis* to ferment mannitol [38]. Additionally, to verify the *E. faecium*/*E. faecalis* discrimination, all enterococcal isolates were grown in M17 broth containing ampicillin (final concentration 2 μg mL^−1^ from Sigma Aldrich), as *E. faecalis* is susceptible to this antibiotic [38]. Arabinose fermentation (1% *w*/*v* from Sigma Aldrich) was used for a preliminary discrimination of *E. faecium*, as *Enterococcus durans* and *Enterococcus hirae* are not able to ferment this monosaccharide [39]. Similarly, *Lacticaseibacillus casei* and *paracasei* were discriminated using mannitol (1% *w*/*v*), which is only fermented by *L. paracasei* [40], while *Leuconostoc mesenteroides*/*paramesenteroides* isolates were differentiated taking into consideration their ability to hydrolyze salicin (1% *w*/*v* from Sigma Aldrich) and aesculin (1% *w*/*v* from Sigma Aldrich), as *L. paramesenteroides* rather fails to hydrolyze both glucosides [41]. Finally, malonic acid (1% *w*/*v* from Sigma Aldrich) was used to discriminate *Hafnia alvei* vs. *paralvei* as it is utilized only by *H. alvei* strains [42].

### 2.7. Antimicrobial Activity of the Isolated Strains

All isolates were tested for their antimicrobial activity against 20 indicator strains, including four LAB strains, 10 pathogenic streptococci, three *Listeria* spp., two *Bacillus* spp. And one *Pseudomonas* sp. The well diffusion assay (WDA) was performed to assess the antimicrobial activity of the cell-free culture supernatants with pH adjusted to 6.5 [43]. Treatment with ammonium sulfate (60% saturation from AppliChem GmbH, Darmstadt, Germany), was applied in selected active supernatants for 10-fold protein concentration and therefore increase of the antimicrobial activity.

## 3. Results and Discussion

### 3.1. Physicochemical Analyses

Physicochemical characteristics of Staka samples, namely pH and titratable acidity, as well as moisture, ash, and fat and protein content, are summarized in Table 1.

The pH values, ranging from 4.6 to 5.3, were similar or higher than those reported for other sour cream samples studied, where the pH ranged between 3.8 and 4.8 [44]. This is an important finding as it has been reported that when the final product pH is very low (e.g., around pH 4.0), the cream has an unpleasant sour flavor [10]. However, the high pH when coupled with high moisture content, as in our study for Staka 1 and 5, may pose spoilage and safety issues and thus shorten the product’s shelf-life. Titratable acidity was low in four samples (Staka 1, 2, 3, and 5), ranging from 0.2 to 0.5% *w*/*w* with the exception of Staka 4. The higher TA of Sample 4 (1.2 ± 0.3% *w*/*w*) is in accordance with the high lactic acid concentration determined (16.3 ± 0.5 g/kg); at the same time, the rather high pH of 5.2 ± 0.2 can be attributed to the buffering capacity of the proteins (16.00 ± 0.78% *w*/*w*), which was the highest observed among samples, most probably due to the addition of wheat flour during processing (cooking). These results are in agreement with reported values for sour cream samples ranging from 0.5 to 1.7% [44].

Moisture and ash values ranged from 27.1 to 48.3% *w*/*w* and from 0.2 to 3.4% *w*/*w*, respectively. Ash values varied from 1.9 to 2.7% *w*/*w* for samples of Ispir Kaymak, although this product is not fermented [16].

Fat content values, ranging between 30.3 and 46.5% *w*/*w*, were consistent with values reported for fat content of cultured creams, such as full-fat commercial sour creams collected from across the US which ranged between 16.8–33.1% *w*/*w* [44], meeting the composition requirements of full fat sour creams, which must have at least either 10% *w*/*v* [45] or 18% *w*/*v* [8,46] milk fat. Concerning the fat content, Staka samples resembled to the Lithuanian crème fraȋche and Ispir Kaymak, which had a fat content around 30–45% [10] and 43–63% [16], respectively. Therefore, Staka is a dairy product with a high fat content that may constitute a rich source of beneficial lipids [47]. Additionally, the fat content expressed as fat in dry matter (FDM) was 64.1 and 56.0%, respectively, for Staka 1 and 3, and these values were consistent to those reported on the labels of the products (min 40.0 and 55.0% *w*/*w*, respectively). Fat content was not reported for the rest of the samples.

Protein content of Staka 1, 2, 3 ranged from 2.3 to 3.0% *w*/*w*, while values for Staka 4 and 5 were significantly higher (16.0%, and 6.4% *w*/*w*, respectively); this can be attributed to the processing (cooking) of these samples with flour, since wheat, no matter wholemeal or not, contains ca. 12% *w*/*w* proteins. However, the difference between Samples 4 and 5 cannot be explained since we did not get any further information from the producers about the ratio of milk cream and flour. High protein values ranging from 14.3 to 20.3% *w*/*w* have been recorded for samples of Ispir Kaymak [16]. Furthermore, Staka 4 had also the lowest fat content. This was due to the removal of the fat content to produce Stakovoutiro (butter) in parallel to Staka. Such a procedure may alter the overall composition of the particular sample and justify its deviation from the rest of the samples in terms of composition.

HPLC results (Table 2) showed that lactic acid was the prominent organic acid in all samples, as also reported for commercial US sour creams [44], followed by acetic acid (Staka 1, 2, 3, 5). Varying concentrations of lactic acid as well as acetic acid depend on the lactose catabolism pathway that the members of the samples’ microbiota follow, i.e., homo- or hetero-fermentative or both.

### 3.2. Culture-Based Microbiological Analysis

Table 3 summarizes the results obtained through culture-based microbiological analysis. Interestingly, Staka 4 and 5 were obviously affected by the heating step in the presence of flour that took place during their production. More specifically, Staka 4 presented low populations of lipolytic bacteria, thermophilic LAB and NSLAB, while none of the microbial groups tested could be detected in Staka 5.

Regarding the rest samples (Staka 1, 2 and 3), which did not receive such treatment, in the case of total mesophilic bacteria as well as the six major groups of presumptive LAB, counts ranging from 6.03 to 8.09 log cfu g^−1^ were observed, and only in Staka 1 lower counts were determined for thermophilic lactobacilli, thermophilic cocci and enterococci (4.51–4.86 log cfu g^−1^). These differences could be attributed to the microbiota of the milk used, the production environment and utensils employed as well as the technology applied. LAB are known as the main actors in dairy fermented foods; comparable LAB counts have been reported for traditional sour cream from Russia [48], Ukraine [18] and Colombia [19].

Analogous counts (6.70–8.05 log cfu g^−1^) were enumerated for lipolytic and proteolytic bacteria. Such bacteria can have either beneficial or detrimental impact on fermented foods, depending on the species of microorganisms and the flavor compounds they produce [49]. For instance, LAB, despite being only weakly lipolytic and proteolytic, play an important role in dairy fermented foods as they shape not only their sensory traits but also their nutritional attributes [50]. Regarding psychrotrophic bacteria, *Pseudomonas* spp. and coliforms, higher counts were detected in Staka 1, followed by Staka 3, while in Staka 2, these populations were absent. *Pseudomonas* spp. have been associated with very high levels of lipase and protease activities that can cause off-flavors [51]. Furthermore, the presence of coliforms, which are indicators of poor hygiene conditions during food processing, has also been reported in artisanal Colombian Suero Costeño sour cream [19].

Yeast populations varied, with the highest value corresponding to Staka 3 (5.50 log cfu g^−1^), followed by Staka 1 and 2 (4.11 and 3.41 log cfu g^−1^, respectively). The presence of yeasts has also been reported in Suero Costeño sour cream with values even higher than 6.0 log cfu mL^−1^ [19]. Sour cream is one of the cultured milk products which is a favorable medium for the propagation of yeasts, since it exhibits relatively low pH values ranging between 4.0 and 6.0 [52].

### 3.3. Amplicon Sequencing and Shotgun Metagenomics

**Figure 1:**
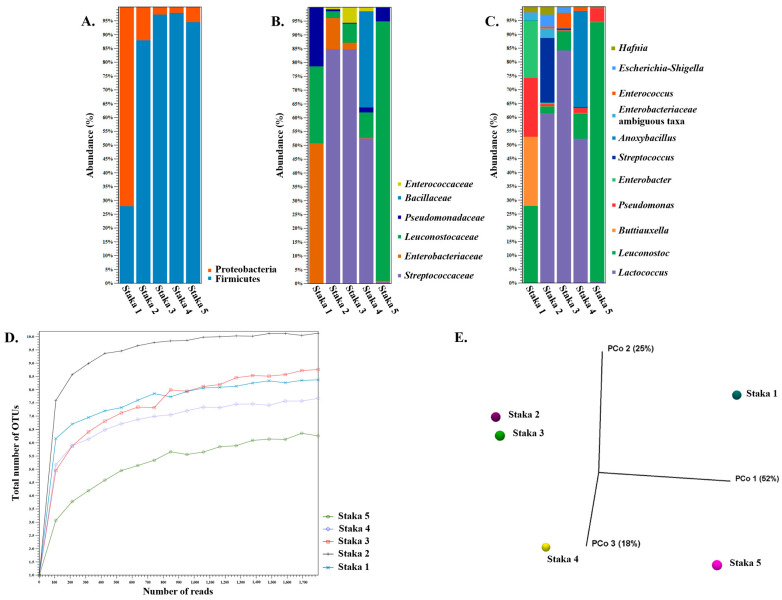
Taxonomic profile of Staka sour cream samples based on 16S rDNA amplicon data at the phylum (**A**), family (**B**), and genus (**C**) levels. Panel (**C**) presents genera with abundance ≥ 1%. Alpha-diversity analysis of 16S rDNA measured using the total number of OTUs at genus level of Staka samples (**D**). Beta-diversity shown through a principal coordinate analysis (PCoA) employing the Bray–Curtis distances for the same samples (**E**).

Figure 1 presents the findings of the 16S rDNA amplicon sequencing analysis of the Staka samples. In all samples, Firmicutes was the major phylum (>88% abundance), followed by Proteobacteria, except for Staka 1, in which the abundance of the two phyla was reversed with Proteobacteria reaching 72%. At the family level, significant differences in bacterial composition across the samples could be observed. *Streptococcaceae* was the dominant family in Staka 2, 3, and 4 with ca. 85% abundances in the first two and a 52% abundance in the third. *Enterobacteriaceae* exhibited the highest abundance of 50% in Staka 1, while *Leuconostocaceae* dominated Staka 5 with a 94% abundance. *Leuconostocaceae* was the second-highest population in Staka 1 (28% abundance) followed by *Pseudomonadaceae* (22% abundance). *Leuconostocaceae* was also present in Staka 2, 3, and 4 but in much lower abundances (<9%). Low levels of *Pseudomonadaceae* also appeared in the rest of the samples with <5% abundance. Furthermore, *Bacillaceae* was present with 35% abundance as the second-largest population in Staka 4, and *Enterococcaceae* appeared at low abundances (<6% abundance) in Staka 2, 3, and 4. The analysis at the genus level demonstrated that Staka 1 was composed of *Leuconostoc* (28% abundance), *Buttiauxella* (25% abundance), *Pseudomonas* (22% abundance), and *Enterobacter* (21% abundance). Staka 2, 3, and 4 were characterized by the high abundance of *Lactococcus* (up to 84%). The second-largest population in Staka 2 was *Streptococcus* (24% abundance), while in Staka 4, it was *Anoxybacillus* (35% abundance). Staka 5 was practically composed of *Leuconostoc* (94% abundance) and *Pseudomonas* (5% abundance). In different samples, several genera were present in variable and relatively low abundances, including *Leuconostoc* (Staka 2, 3, and 4, <9% abundance), *Pseudomonas* (Staka 2, 3, and 4, <2% abundance), *Enterococcus* (Staka 2, 3, and 4, <6% abundance), *Enterobacteriaceae* ambiguous taxa (Staka 1 and 2, <4% abundance), *Escherichia*-*Shigella* (Staka 1, 2, and 3, <4% abundance), and *Hafnia* (Staka 1 and 2, <3% abundance).

Alpha-diversity of total number of genus level operational taxonomic units (OTUs) indicated that all samples were sequenced to a sufficient depth (Figure 1D). Staka 2 had the most complex microbiome composition, while Staka 5 had the simplest one. The complexity of the microbiomes of Staka 1, 3, and 4 seemed to be relatively comparable. In addition, beta-diversity could segregate Staka 2, 3, and 4 from Staka 1 and 5 in a principal coordinate analysis (PCoA) using Bray–Curtis distances (Figure 1E). This could be attributed to the dominance of the genus *Lactococcus* in Staka 2, 3, and 4 versus the dominance of *Leuconostoc* accompanied by the absence of *Lactococcus* in Staka 1 and 5.

The findings of the analysis of the 16S rDNA amplicons support the presence of LAB, *Enterococcus* sp., coliforms (e.g., *Buttiauxella* sp., *Enterobacter* sp., *Escherichia* or *Shigella* sp. and *Hafnia* sp.), and psychrotrophs (e.g., *Pseudomonas* sp.) as also determined by the initial culture-based microbiological analysis presented above. Several of these groups/genera may contain strains that are lipolytic and/or proteolytic. It should be highlighted that both Staka 4 and 5 provided 16S rDNA fingerprints, even though the ecosystem of both samples was seriously affected by the heat treatment step employed during their productions. Several bacterial OTUs could be assigned to Staka 4 and 5, even though the first presented very low microbial populations and the second seemed to be sterile. Seemingly, DNA of dead cells or unculturable cells was retained, and thus, 16S rDNA amplicon sequencing could provide retrospective information of the original microbiome composition. In addition, the distribution of genus level OTUs with >1% abundance did not always correlate with that of the culture-based microbiological analysis. For example, enterococci were present in the first three samples (Table 3), but they seemed to be practically absent from the OTUs in Staka 1. This may be an effect of the different populations of enterococci among the samples. As already mentioned, in Staka 1 enterococci had their lowest population ca. 2–3 log lower than their population in Staka 2 and 3 (Table 3), and thus, they may not be presented in the genus level OTUs with >1% abundance. Our findings are partially in agreement with those reported for Suero Costeño [19]. While several bacterial genera are in common between Staka and the Colombian sour cream (e.g., *Lactococcus*, *Leuconostoc*, *Enterobacter*, *Escherichia*-*Shigella*, etc.), the most abundant OTUs reported for samples of the latter were *Lactobacillus* or *Streptococcus*. This is a major difference of our samples, which were dominated by *Lactococcus* or *Leuconostoc*. Interestingly, in the Staka samples analyzed, lactobacilli were only present with abundances <1%. A prevalence of *Lactococcus* or *Streptococcus* accompanied with a low abundance of lactobacilli has also been reported for Russian sour cream products [23,53]. Of note, *Acetobacter* sp. were reported for both Suero Costeño and some of the Russian sour cream, but they were absent from the Staka samples.

**Figure 2:**
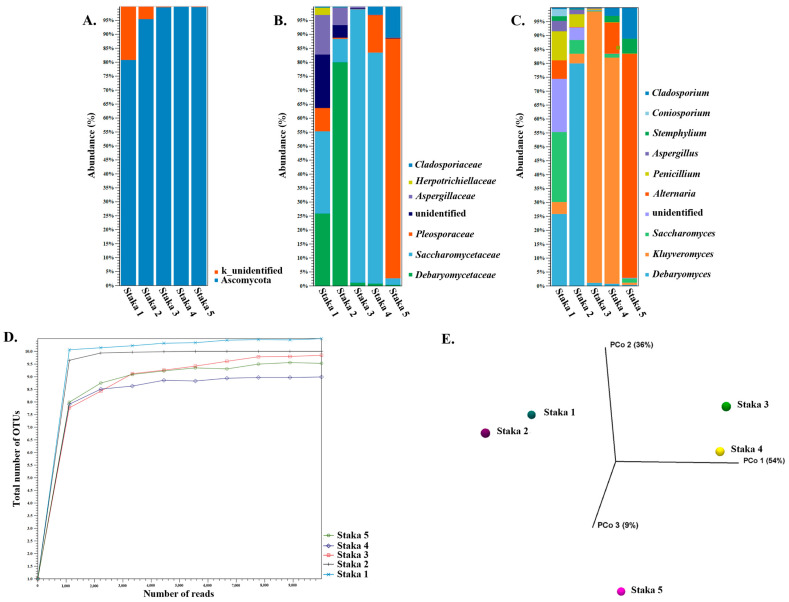
Taxonomic profile of Staka sour cream samples based on ITS amplicon data at the phylum (**A**), family (**B**), and genus (**C**) levels. Panel (**C**) presents genera with abundance ≥ 1%. Alpha-diversity analysis of ITS reads measured using the total number of OTUs at genus level of Staka sour cream samples (**D**). Beta-diversity shown through a principal coordinate analysis (PCoA) employing the Bray–Curtis distances for the same samples (**E**).

All Staka samples were also analyzed using ITS amplicon sequencing (Figure 2). Ascomycota was the prevailing phylum in all samples with an abundance >81%, reaching up to >99% in Staka 3, 4, and 5 (Figure 2A). With the exception of Staka 4, an additional population unidentified at kingdom level (k_unidentified) was also present in all samples, with abundances reaching 19% in Staka 1 and 4% in Staka 2. Manual analysis with BLASTn indicated that the relevant ITS sequence belongs to the ascomycete fungi *Iodophanus* sp., which could not be identified as such by the Unite database used in this study. At the family level, *Saccharomycetaceae* was the predominant family in Staka 1, 3, and 4 with abundances of 29%, 98%, and 83%, respectively. The *Saccharomycetaceae* family was the second largest in Staka 2 with an abundance of 8% and was also present in Staka 5 with a 3% abundance. In contrast, the most abundant family for Staka 2 was *Debaryomycetaceae* (81% abundance) and *Pleosporaceae* (86% abundance) for Staka 5. The *Debaryomycetaceae* family was the second largest in Staka 1 (26% abundance) and was also found in low abundances in Staka 3, 4, and 5, reaching up to 1% in Staka 3. The *Pleosporaceae* family formed the second largest population in Staka 4 (13% abundance) and was also found in the rest of the samples, reaching an abundance of 9% in Staka 1. Other fungi present in the Staka samples were members of the *Aspergillaceae* family found in Staka 1, 2, and 3 with abundances equal to 14%, 7%, and 1%, respectively. Additionally, the *Cladosporiaceae* family formed the second-largest population in Staka 5 with an 11% abundance, and it was also identified in Staka 1, 2, and 4, reaching up to 3% abundance. The *Herpotrichiellaceae* family was present only in Staka 1 with an abundance of 3%. Except for Staka 4, in all samples the unidentified kingdom mentioned above corresponded to the *Pezizaceae* family (determined manually), reaching 19% abundance in Staka 1. We then performed analysis to establish the prevailing genera with >1% abundance in all samples (Figure 2C). Some of the families described above had only one representative genus, i.e., the *Debaryomyces* for the *Debaryomycetaceae* family, *Coniosporium* for the *Herpotrichiellaceae*, and *Cladosporium* for the *Cladosporiaceae* family. The *Saccharomycetaceae* family was present with genera *Kluyveromyces* and *Saccharomyces*, the *Pleosporaceae* family consisted of *Alternaria* and *Stemphylium* genera, and the *Aspergillaceae* family included the *Penicillium and Aspergillus* genera. As mentioned above, the unidentified genus could be matched to *Iodophanus* sp. of the *Pezizaceae* family.

Alpha-diversity of the total number of genus level OTUs showed that a sufficient sequencing depth of all samples was reached. Beta-diversity based on the genus level using Bray–Curtis distances showed some similarity between pairs of Staka 1 and 2 and Staka 3 and 4, while Staka 5 was found separate from the rest. This could be attributed to the presence of *Debaryomyces* in Staka 1 and 2, *Kluyveromyces* in Staka 3 and 4, and *Alternaria* in Staka 5.

Unfortunately, information about the presence of yeasts or molds in sour cream seems to be rare. *Candida* sp., *Rhodotorula* sp., and *Cryptococcus* sp. have been detected in pasteurized cream (non-sour) [54]. Some studies have clearly indicated that yeast and molds are related to the spoilage of sour cream [55,56]. These microorganisms may originate from raw milk, but they may be also characteristic of the dairy facility. Almost all of the genera identified in our samples are more or less common in fermented dairy products, e.g., *Debaryomyces*, *Kluyveromyces*, *Saccharomyces*, *Alternaria*, *Cladosporium*, *Penicillium,* and *Aspergillus* [57,58,59,60]. Three genera, i.e., *Iodophanus*, *Cladosporium,* and *Stemphylium,* seem to be rather rarely related to the dairy environment [61,62,63], and thus their presence needs further investigation. It should be noted that the uncontrolled presence of genera like *Penicillium* and *Aspergillus* may be problematic, given their ability to produce mycotoxins that can remain unaffected during normal production and storage conditions [59].

**Figure 3:**
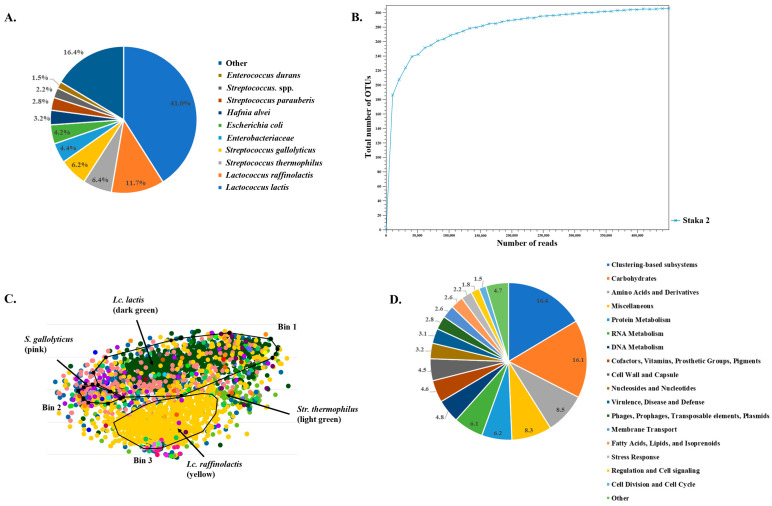
Taxonomic profile of Staka 2 sour cream sample, based on the mapping of shotgun metagenomics reads at the species level (**A**). Alpha-diversity of shotgun reads measured using the total number of OTUs of Staka 2 (**B**). Bins of metagenomics scaffolds of Staka 2 (**C**). Dots of same colors represent scaffolds that originate from the same species, as indicated in the figure. Functional analysis of annotated scaffolds of Staka 2 using the MG-RAST server (**D**).

Staka 2 was chosen for further investigation of the Staka microbiome with shotgun metagenomics, given the high complexity it showed in the alpha-diversity of both 16S rDNA and ITS OTUs. The analysis revealed the dominance of members of the *Streptococcaceae* family, i.e., *Lc. lactis* with an abundance of 41%, *Lc. raffinolactis* with an abundance of 11.7%, and each of *S. thermophilus* and *S. gallolyticus* with abundances of ca. 6% (Figure 3A). The unidentified species of the *Enterobacteriaceae* family and *Escherichia coli* were present with abundances of 4.4% and 4.2%, respectively. *H. alvei* showed an abundance of 3.2%, followed by *Streptococcus parauberis* with an abundance equal to 2.8%. Unidentified *Streptococcus* spp. and *E. durans* were present with populations of 2.2% and 1.5%, respectively. Other species present in Staka 2 occupied 16.4% abundance in total. Alpha-diversity suggested a sufficient sequencing depth, while the taxonomic findings of the shotgun analysis of Staka 2 are in overall agreement with the 16S rDNA amplicon sequencing results (Figure 3B). The absence of fungi from the shotgun analysis may be due to their low population in the sample as mentioned above.

**Figure 4:**
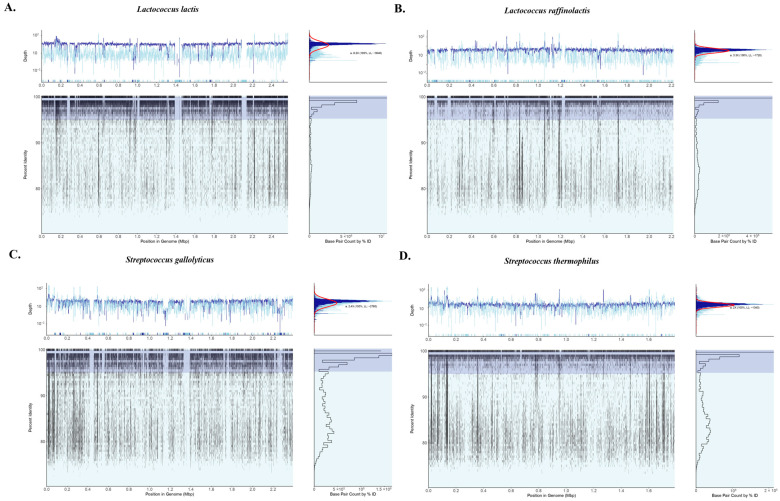
Recruitment plots of the sequencing reads of Staka 2 sour cream sample against reference genomes of *Lactococcus lactis* (CP015902.1) (**A**), *Lactococcus raffinolactis* (CP023392.1) (**B**), *Streptococcus gallolyticus* (CP113954.2) (**C**), and *Streptococcus thermophilus* (CP031545.1) (**D**).

To identify putative metagenome-assembled genomes (MAGs), contigs from the assembled reads of Staka 2 were further analyzed with the BusyBee server, and three bins were determined (Figure 3C). The quality of the bins was not sufficient, but the taxonomic analysis could assign the majority of contigs in Bin 1 and Bin 3 to *Lc. lactis* and *Lc. raffinolactis*, respectively. Several contigs in Bin 2 could be assigned to *S. gallolyticus*, while contigs assigned to *S. thermophilus* were relatively grouped together but did not form a separate bin. To further aid the identification of chromosomal sequences in the dataset, at least for the four most abundant species in the sample analyzed, and we employed recruitment plots of the sequencing reads against reference genomes. As can be seen in Figure 4, almost full draft genomes could be formed during the recruitment of the reads against each of the reference genomes with relatively short gaps. In fact, an important number of reads could be aligned with an identity > 95% despite the fact that the overall coverage was low, ranging from 8.2× for *Lc. lactis* (the most abundant species) to 2× for *S. thermophilus* (the least abundant species). Thus, recruitment plot analysis provided more comprehensive results than the binning of the contigs.

As mentioned above, *Lc. lactis* can be used as a starter for the acidification of the cream [10]. *Lc. lactis* was also present in the full-length 16S rRNA amplicon sequencing of sour cream collected in northeast Asia [53]. *Lc. raffinolactis* has also been reported as a member of the microbiome of sour cream [53]. *S. thermophilus* has been detected in the previous study of Staka [25], but its presence has been verified in other sour creams as well [48]. *Enterobacteriaceae* are frequent member of the microbiome of artisanal dairy products, and some genera of the family have been identified in sour cream [19,53]. To the best of our knowledge, *S. gallolyticus*, *H. alvei*, and *S. parauberis* are reported for the first time in sour cream. Even though the presence of enterococci in sour cream has been reported, *E. durans* has been identified again only in Staka in the past [25].

In order to shed light on the functional potential of the Staka microbiome, functional analysis of the assembled shotgun metagenomes of Staka 2 was performed using the MG-RAST server. Scaffolds were annotated and annotations were assigned to functions. As seen from the Figure 3D, the main part (16.4%) of the Staka 2 functional subsystems belonged to clustering-based subsystems, but their role in the metabolic pathways is yet unknown. Among other subsystems assigned were carbohydrates which occupied the second place with 16.1%, followed by such subsystems as amino-acids and derivatives (8.5%), miscellaneous (8.3%), protein metabolism (6.2%), RNA metabolism (6.1%), and DNA metabolism (4.8%). Additional functional categories could also be identified with decreasing percentages.

### 3.4. Isolates Typing and Identification

A total of 141 bacterial isolates were collected from the five Staka samples and identified. Based on rep-PCR analysis of bacterial strains, they were clustered in 49 groups (Supplementary Table S1). Representative isolates of all groups were selected and subjected to 16S rDNA sequencing. According to the results obtained, 78 isolates were identified as *Enterococcus* spp., 21 as *Leuconostoc mesenteroides*/*pseudomesenteroides*, 13 as *Hafnia alvei*/*paralvei*, 5 as *Pseudomonas* spp., 6 as *Bacillus subtilis*, 6 as *Serratia liquefaciens*, 5 as *Lacticaseibacillus casei*/*paracasei* (basonym *Lactobacillus casei*/*paracasei*), 3 as *Companilactobacillus versmoldensis* (basonym *Lactobacillus versmoldensis*), 2 as *Latilactobacillus curvatus* (basonym *Lactobacillus curvatus*), 1 as *Loigolactobacillus coryniformis* (basonym *Lactobacillus coryniformis*), and 1 as *S. thermophilus*.

It should be stressed, however, that 16S rDNA gene sequencing cannot discriminate *L. casei* from *L. paracasei* [64]. The same is valid for closely related enterococcal species, i.e., *E. faecalis*, *E. faecium*, *E. durans* and *E. hirae* [65], *L. mesenteroides* and *L. pseudomesenteroides* [66], and *H. alvei* and *H. paralvei* [42], as well as *Pseudomonas* species [67]. To overcome this obstacle and identify the enterococcal isolates at the species level, mannitol and arabinose fermentation, as well as the ampicillin resistance of the isolates, were considered. According to the results obtained, 24 isolates were identified as *E. faecalis* as they fermented mannitol and were ampicillin susceptible, while 54 were identified as *E. faecium* as they did not ferment mannitol, while they fermented arabinose. Respectively, all *L. casei*/*paracasei* isolates were identified as *L. paracasei* as they fermented mannitol, all *L. mesenteroides*/*pseudomesenteroides* isolates were identified as *L. pseudomesenteroides* as they could not hydrolyze salicin and aesculin, and finally, all *Hafnia alvei*/*paralvei* isolates were identified as *Hafnia paralvei* as they did not use malonic acid. Finally, the presence of Gram-negative and Gram-positive spoilage or opportunistic pathogenic bacteria, i.e., *Pseudomonas* spp. (five isolates in Staka 1), *Serratia liquefaciens* (six isolates in Staka 1), and *B. subtilis* (six isolates in Staka 4) were detected.

Interestingly, no *Lactococcus* spp. could be isolated from the samples during culture-based analysis, in contrast to *Leuconostoc* spp. and *Enterococcus* spp., which were readily isolated. Several isolates, also belonging to the genus *Enterococcus*, were detected in the Staka sample examined by Lappa et al. (2021). In addition, Lappa et al. (2021) reported the isolation of *L. paracasei*, *Lactiplantibacillus plantarum* (basonym *Lactobacillus plantarum*), and *Lactiplantibacillus paraplantarum* (basonym *Lactobacillus paraplantarum*). Clearly, findings about the culturable fraction of the microbial ecosystem of the aforementioned study and the present study cannot be directly correlated with those of the amplicon sequencing and shotgun metagenomics analysis. There are several different putative reasons which may underpin such differences. First of all, colonies in both studies were not picked randomly but on the basis of differences in morphology, and thus, the frequency of taxa defined after cultured-based analysis may not be correlated to that defined after culture independent metagenomics analysis. Second, the conditions prevailing during cell culture may have favored the growth and the subsequent selection of a fraction of the species present in the microbiome of the Staka samples. Finally, the inability to isolate certain species exhibiting high abundances in the Staka samples in silico may indicate that at least some of them could have entered a viable but not culturable state (VBNC) during production and/or storage. Such a scenario is plausible, and there are important data to support it as in the case of *Lc. lactis* [68,69,70,71]. In detail, three strains of *Lc. lactis* used to produce a model cheese could be identified as viable by RT-qPCR but could not be recovered by traditional plating on M17 medium through the ripening period [70]. *Lc. lactis* can enter a VBNC state under carbohydrate starvation [72], which may prevail during ripening and/or storage of dairy products. In another study concerning the dynamics of LAB during long-term ripening of cheddar investigated by culture-based analysis, qPCR and 16S rDNA amplicon sequencing, the accumulation of a stable population of *Lactococcus* spp.-permeable cells was demonstrated after treatment of cells with propidium monoazide (PMA) [69]. PMA intercalates in the DNA of cells with membrane damage and inhibits its amplification by PCR. This population was not able to form colonies. Moreover, the presence of *Lc. lactis* cells in the VBNC state was verified in some traditional Lebanese products [68]. While *Lc. lactis* was identified in the metagenomics of some samples, it was not possible to isolate it under typical M17 isolation conditions. Most importantly, adding goat milk in the medium allowed the recovery of *Lc. lactis* colonies, verifying the original VBNC state of these cells. Furthermore, different *Enterococcus* spp. could be identified in the shotgun data of Staka 2 and in the 16S rDNA of the other samples. The same also applies for *Hafnia* spp. These may explain the isolation of *E. faecium* and *E. faecalis*, as well as *H. paralvei*, rather than *E. durans* and *H. alvei*, which were predicted through metagenomics analysis. Similar and even more major deviations between culture-based analysis and metagenomics seem not to be uncommon (Papadimitriou et al. unpublished results; [71]).

### 3.5. Antimicrobial Activity

All bacterial isolates were examined for antimicrobial activity against 20 indicator strains. Cell-free culture supernatants (CFCSs) of milk as well as the MRS broth (bacilli) or M17 broth (cocci) of overnight cultures were tested. The results for all isolates are summarized in Supplementary Table S2. Positive results concerning the supernatants of selected isolates exhibiting antimicrobial activity, which were further 10-fold concentrated, are presented in Table 4.

Regarding antimicrobial activity against Gram-negative indicators, very low or borderline inhibition was detected against *Pseudomonas aeruginosa* FMCC B-26 by a few strains, namely *L. paracasei* ACA-DC 1119, *L. pseudomesenteroides* ACA-DC 1145, and *Enterococcus faecium* ACA-DC 1117, 1200, 1201, and 1216. *P. aeruginosa* strains are considered responsible for an increased number of nosocomial infections incidents caused by Gram-negative multi-drug resistant (MDR) bacteria [73]; therefore, these, even with low antimicrobial activities, deserve further study so as to clarify if they correspond to bacteriocins. Bacteriocin L-1077 and Enterocin E-760 produced by *Ligilactobacillus salivarius* (basonym *Lactobacillus salivarius*) and *E. faecalis*, respectively, have been previously reported to be active against *P. aeruginosa* [74,75].

Regarding Gram-positive target strains, almost all isolates inhibited the growth of at least one LAB target strain. *S. thermophilus* ACA-DC 4 was found to be the most sensitive one, followed by *Latilactobacillus sakei* (basonym *Lactobacillus sakei*) ACA-DC 2313. *L. curvatus* ACA-DC 1135, along with three *L. pseudomesenteroides* isolates, namely ACA-DC 1128, 1131, and 1249, were found active against the pathogenic *Streptococcus pneumoniae* LMG 14545^T^. *L. paracasei* ACA-DC 1260 and *C. versmoldensis* ACA-DC 1262 were active against *Streptococcus oralis* LMG 14532^T^. Two strains, namely *L. coryniformis* ACA-DC 1251 and *L. pseudomesenteroides* ACA-DC 1130, were active against both the above pathogenic streptococci. The above-mentioned species have been previously reported to produce bacteriocins, e.g., curvaticin L442 [76], leucocyclicin Q [77], and reuterin from *L. coryniformis* [78]. The antimicrobial activity of the isolated NSLAB strains against pathogenic streptococci reinforces the suitability for their use as adjunct starter cultures and/or nutraceuticals. To our knowledge, there is no report on bacteriocin characterization from *C. versmoldensis*; however, it has been included in a comparative genomics study where bacteriocin production was predicted with bioinformatics tools [79]. Among the isolated enterococci, 13 were found to have antilisterial activity, 10 were active against at least one pathogenic streptococcal isolate (mostly *S. oralis* LMG 14532^T^), and among them, 3 inhibited one pathogenic *Streptococcus* sp. strain (*S. oralis* LMG 14532^T^ or *Streptococcus sanguinis* DSM 20068) along with one or three *Listeria* spp. strains.

Finally, various non-LAB isolates, namely *H. paralvei*, *B. subtilis* and *Pseudomonas* spp., exhibited antimicrobial activity against pathogenic streptococcal and *Listeria* species. Interestingly, three *Pseudomonas* spp. isolates inhibited from three to ten pathogenic streptococci, while two inhibited *B. cereus* LMG 6923^T^ and *B. subtilis* FMCC B-109 strains and/or the *L. welshimeri* 15008. This antimicrobial activity can be attributed either to antibiotic production, as antibiotics like phenazine-1-carboxylic acid and mupirocin are produced by *Pseudomonas* sp. [80], or to the so-called pyocins, the bacteriocins produced by *P. aeuroginosa* [81]. Pyocins have been previously reported to possess activity against Gram-positive bacterial species, e.g., pyocin SA189 against *S. aureus*, *Streptococcus pyogenes*, and *L. monocytogenes* [82], and pyocin RPU15 against *S. aureus*, *L. monocytogenes*, and *Bacillus cereus* [83]. These results need to be further elucidated, since antibiotics are not impactful anymore against the MDR pathogens’ threat, and new approaches are gaining ground to produce food antibacterial compounds.

## 4. Conclusions

Staka is a traditional Greek sour cream produced in Crete. Based on the physicochemical analysis, it may have a slightly higher pH compared to other sour creams. Its titratable acidity aligns with similar dairy products, indicating a mild sourness. The relatively increased moisture content along with high values of pH could negatively affect its safety and shelf-life. The high fat content, like that of other creamy dairy products, contributes to its mouthful and its potential as a source of beneficial lipids. The protein content varies, especially if Staka is processed with flour, affecting texture and nutritional value. In all samples analyzed, lactic acid was the predominant organic acid detected, while other acids were present in minor concentrations. The culture-based microbiological analysis of Staka revealed how processing variations could influence microbial populations. Heating (with or without flour) may significantly reduce detectable microbes, even leading to no detectable counts. Amplicon sequencing, both 16S rDNA and ITS analyses, provided a comprehensive view of the Staka microbiome concerning bacterial and fungal communities. As mentioned above, the production process affected microbial diversity, as certain heat-treated Staka samples showed reduced or absence of culturable microbial populations. Despite this, amplicon sequencing captured DNA from non-viable or unculturable cells, offering insight into the original microbiome. Shotgun metagenomics analysis of Staka provided a detailed picture of its microbiome, revealing a diverse community predominantly composed of *Streptococcaceae* members like *Lc. lactis* and *Lc. raffinolactis*. The construction of some near-complete MAGs, with both the binning of assembled contigs and reference-based recruitment plots analysis of single reads, further refined the understanding of the microbiome, assigning a majority of contigs and reads to specific bacterial species, enhancing the specificity of microbial identification. Subsequently, isolated bacterial strains were screened for their antimicrobial potential. Most isolates inhibited at least one LAB strain, with various isolates being also active against oral or other pathogenic *Streptococcus* species. Inhibition against Gram-negative *P. aeruginosa* was generally low, but some isolates showed potential for further study due to their antimicrobial activity against this indicator. Additionally, non-LAB isolates like *H. paralvei*, *B. subtilis*, and *Pseudomonas* spp. showed broad antimicrobial activity, possibly due to their ability to produce antibiotics or bacteriocins. These findings highlight that at least some of the isolates could be valuable as starter cultures or as nutraceuticals. Overall, the findings of our study contribute to a deeper knowledge of the microbial ecosystem in Staka, revealing potential influences on its quality, safety, and sensory attributes. Further studies employing multi-omics approaches may shed light on the relation between the microbiome and metabolome of Staka cream which may affect its physicochemical and organoleptic characteristics. In such studies, the cooking step with flour, wholemeal or not, should also be considered since it may have an important effect on the quality of the final product.

## Figures and Tables

**Table 1:** Description and physicochemical characteristics of Staka samples. Values presented are the means ± SD (n = 3).

					Concentration (% *w*/*w*)					
Staka Sample	Production	Geographical Origin ^c^	Milk Type	pH	TA ^f^	Moisture	Dry Matter	Ash	Fat	Protein
1	C ^a^	Sfakia, Chania	S ^d^	5.2 ± 0.2	0.3 ± 0.1	34.52 ± 1.5	65.48 ± 1.5	0.27 ± 0.07	41.87 ± 0.90	2.31 ± 0.40
2	C	Keramia, Chania	S	4.6 ± 0.3	0.4 ± 0.1	29.54 ± 1.9	70.46 ± 0.9	0.21 ± 0.04	46.47 ± 0.74	2.62 ± 0.15
3	C	Varypetro, Chania	S & G ^e^	4.7 ± 0.4	0.5 ± 0.1	36.43 ± 0.8	63.57 ± 2.0	0.22 ± 0.03	40.57 ± 0.88	3.00 ± 0.25
4	H-M ^b^	Palaiokastro, Sitia	S & G	5.2 ± 0.2	1.2 ± 0.3	27.05 ± 1.1	72.95 ± 1.7	3.42 ± 0.49	32.68 ± 0.66	16.00 ± 0.78
5	C	Chamezi, Sitia	S & G	5.3 ± 0.1	0.2 ± 0.1	48.25 ± 0.7	51.75 ± 1.2	2.15 ± 0.31	30.31 ± 1.70	6.40 ± 0.11

^a^ Commercial; ^b^ homemade; ^c^ Crete perfecture; ^d^ sheep; ^e^ goat; ^f^ titratable acidity expressed in lactic acid concentration.

**Table 2:** Concentration of lactose, organic acids and ethanol detected in Staka samples as determined by HPLC analysis. Values presented are the means ± SD (n = 3).

	Concentration (g/kg)						
Staka Sample	Lactose	Lactic Acid	Acetic Acid	Succinic Acid	Butyric Acid	Propionic Acid	Ethanol
1	24.7 ± 0.4	8.1 ± 0.5	1.4 ± 0.4	0.3 ± 0.1	nd	nd	0.4 ± 0.2
2	17.2 ± 0.3	6.7 ± 0.5	1.0 ± 0.2	0.3 ± 0.2	0.1 ± 0	nd	nd
3	18.5 ± 0.3	10.0 ± 0.6	0.8 ± 0.2	nd	nd	nd	0.3 ± 0.2
4	40.0 ± 1.1	16.3 ± 0.5	nd	nd	1.3 ± 0.2	1.7 ± 0.3	nd
5	13.6 ± 0.6	2.0 ± 0.3	1.6 ± 0.3	0.1 ± 0.1	nd	nd	0.2 ± 0.1

nd: not detected.

**Table 3:** Microbial counts (log cfu g^−1^) of the Staka samples examined. Values presented are the means ± SD (n = 3).

	Staka Sample				
Presumptive Microbial Group	1	2	3	4	5
Total mesophilic bacteria (PCA agar, 30 °C)	8.09 ± 0.09	7.10 ± 0.18	7.88 ± 0.26	0.00	0.00
Thermophilic lactobacilli (MRS agar pH 5.4, 42 °C)	4.51 ± 0.35	6.13 ± 0.17	7.45 ± 0.21	0.00	0.00
Mesophilic lactobacilli (MRS agar pH 5.4, 22 °C)	7.99 ± 0.11	6.88 ± 0.39	7.33 ± 0.22	0.00	0.00
Thermophilic cocci (M17 agar, 42 °C)	4.86 ± 0.19	6.18 ± 0.28	7.67 ± 0.42	3.80 ± 0.22	0.00
Mesophilic cocci (M17 agar, 22 °C)	8.14 ± 0.17	6.44 ± 0.81	7.60 ± 0.19	0.00	0.00
NSLAB (Rogosa agar, 30 °C)	7.84 ± 0.41	6.92 ± 0.16	7.52 ± 0.31	2.80 ± 0.23	0.00
Enterococci (KAA agar, 37 °C)	4.70 ± 0.19	6.03 ± 0.22	7.29 ± 0.26	0.00	0.00
Lipolytic bacteria (PCA-tributyrin, 30 °C)	8.05 ± 0.08	6.97 ± 0.10	7.91 ± 0.04	2.60 ± 0.08	0.00
Proteolytic bacteria (PCA-milk, 30 °C)	7.78 ± 0.35	6.70 ± 0.17	7.95 ± 0.59	0.00	0.00
Psychrotrophic bacteria (PCA, 7 °C)	7.35 ± 0.46	4.13 ± 0.09	5.92 ± 0.16	0.00	0.00
*Pseudomonas* spp. (Pseudomonas agar base, 30 °C)	7.72 ± 0.25	0.00	5.85 ± 0.30	0.00	0.00
Coliforms (VRBL agar, 37 °C)	7.54 ± 0.34	0.00	5.60 ± 0.26	0.00	0.00
Yeasts and molds (YGC agar, 25 °C)	4.11 ± 0.26	3.41 ± 0.40	5.50 ± 0.35	0.00	0.00

**Table 4:** Antimicrobial activity of 10-fold concentrated supernatants of selected LAB Staka strains.

Producer Strain ACA-DC Number	Target Strain (Growth Medium of the Producer Strain—mm Inhibition Zone)								
	*S. mutans* LMG 14558^T^	*S. oralis* LMG 14532^T^	*S. pneumoniae* LMG 14545^T^	*S. agalactiae* LMG 14694^T^	*S. salivarius* LMG 11489^T^	*S. sanguinis* DSM 20068	*S. sobrinus* LMG 14641^T^	*S. gordonii* LMG 14518^T^	*S. anginosus* LMG 14502^T^
*L. curvatus* 1135	MRS-10	mye-7t	MRS-10 mye -9	nd	nd	MRS-15	nd	MRS-15	MRS-13
*L. coryniformis* 1251	nd	nd	MRS-9 mye-7	nd	nd	nd	nd	nd	
*L. paracasei* 1260	nd	nd	MRS-11	nd	nd	mye-9t	nd	nd	MRS-10 mye-bl
*C. versmoldensis* 1262	MRS-10	nd	MRS-13	nd	nd	nd	nd	nd	MRS-10
*L. pseudomesenteroides* 1130	mye-10	mye15	MRS-20 mye-15	MRS-8t mye-6t	mye-7t	mye-20	mye-11	mye-20	MRS-14

bl: border line inhibition, t: turbid inhibition zone, mye: milk supplemented with yeast extract, nd: not detected.

## Data Availability

The data presented in this study are openly available in SRA under BioProject IDs: PRJNA1063285, PRJNA1064923, PRJNA1067114.

## Supplementary Materials

The following supporting information can be downloaded at: https://www.mdpi.com/article/10.3390/foods13071129/s1, Supplementary Table S1: Bacteria fingerprinting and identification; Supplementary Table S2: Antimicrobial activity of the Staka bacterial isolates against 20 indicator strains.
