# Supplementary material for: Study of the Microbiome of the Cretan Sour Cream Staka Using Amplicon Sequencing and Shotgun Metagenomics and Isolation of Novel Strains with an Important Antimicrobial Potential
Source: Foods. 2024 Apr 8;13(7):1129. doi: 10.3390/foods13071129 (PMC11011300; doi:10.3390/foods13071129)
Supplement: Supplementary file 1 [file foods-13-01129-s001.zip › foods-2860998-supplementary.pdf]

Supplementary Table S1. Bacteria fingerprinting and identification

**Bacterial Rep-PCR Cluster**	**Identification by 16S rDNA sequencing**	**Number of isolates**	**Staka sample**
C1	*Leuconostoc pseudomesenteroides*	5	2, 3
C2	*Pseudomonas* spp.	1	1
C3	*Leuconostoc pseudomesenteroides*	4	1, 2, 3
C4	*Leuconostoc pseudomesenteroides*	1	2
C5	*Leuconostoc pseudomesenteroides*	3	1
C6	*Leuconostoc pseudomesenteroides*	1	3
C7	*Enterococcus faecium*	1	1
C8	*Leuconostoc pseudomesenteroides*	1	2
C9	*Leuconostoc pseudomesenteroides*	4	1
C10	*Pseudomonas* spp.	2	1
C11	*Enterococcus faecium*	1	2
C12	*Enterococcus faecium*	10	1, 2
C13	*Enterococcus faecium*	15	1, 2
C14	*Enterococcus faecium*	6	2
C15	*Enterococcus faecalis*	4	3
C16	*Enterococcus faecalis*	2	2, 3
C17	*Enterococcus faecalis*	2	3
C18	*Enterococcus faecalis*	1	3
C19	*Lacticaseibacillus paracasei*	5	2, 3, 4
C20	*Enterococcus faecalis*	3	4
C21	*Leuconostoc pseudomesenteroides*	1	3
C22	*Enterococcus faecalis*	2	3
C23	*Enterococcus faecium*	1	3
C24	*Enterococcus faecalis*	1	3
C25	*Enterococcus faecalis*	1	3

C26	*Hafnia paralvei*	6	3
C27	*Enterococcus faecium*	6	3
C28	*Enterococcus faecium*	7	2, 3
C29	*Enterococcus faecalis*	2	3
C30	*Leuconostoc pseudomesenteroides*	1	1
C31	*Hafnia paralvei*	1	1
C32	*Enterococcus faecalis*	1	2
C33	*Serratia liquefaciens*	1	1
C34	*Serratia liquefaciens*	5	1
C35	*Enterococcus faecium*	7	1, 3
C36	*Pseudomonas* spp.	2	1
C37	*Loigolactobacillus coryniformis*	1	2
C38	*Bacillus subtilis*	6	4
C39	*Latilactobacillus curvatus*	1	2
C40	*Hafnia paralvei*	1	1
C41	*Streptococcus thermophilus*	1	2
C42	*Companilactobacillus versmoldensis*	3	4
C43	*Hafnia paralvei*	1	3
C44	*Enterococcus faecalis*	2	3
C45	*Latilactobacillus curvatus*	1	2
C46	*Enterococcus faecalis*	1	3
C47	*Hafnia paralvei*	4	1, 3
C48	*Enterococcus faecalis*	1	3
C49	*Enterococcus faecalis*	1	1

Supplementary Table S2. Antimicrobial activity of the Staka bacterial isolates against 20 indicator strains

Producer strain ACA-DC number	Target strain (growth medium-mm inhibition zone)		
	**LAB**	**Pathogenic streptococci**	**Other spoilage and pathogenic bacteria**
L. paracasei 1119	nd	nd	*P. aeruginosa* FMCC B-26 (mye-bl)
L. curvatus 1135	*S. thermophilus* ACA-DC 4 (MRS-11)	*S. pneumoniae* LMG 14545^T (MRS-9)	nd
L. paracasei 1260	*L. sakei* ACA-DC 2313 (MRS-9);<br>*S. thermophilus* ACA-DC 4 (MRS-12)	*S. oralis* LMG 14532^T (MRS-8t)	nd
C. versmoldensis 1262	*S. thermophilus* ACA-DC 4 (MRS-12)	*S. oralis* LMG 14532^T (MRS-9t)	nd
L. coryniformis 1251	*S. thermophilus* ACA-DC 4 (MRS-12)	*S. oralis* LMG 14532^T (MRS-7t);<br>*S. pneumoniae* LMG 14545^T (MRS-8t, mye-10t)	nd
L. pseudomesenteroides 1128	*S. thermophilus* ACA-DC 4 (MRS-11)	*S. pneumoniae* LMG 14545^T (MRS-9t)	nd
L. pseudomesenteroides 1130	*S. thermophilus* ACA-DC 4 (MRS-11)	*S. oralis* LMG 14532^T (MRS-9t);<br>*S. pneumoniae* LMG 14545^T (MRS-9)	nd
L. pseudomesenteroides 1131	*S. thermophilus* ACA-DC 4 (MRS-11)	*S. pneumoniae* LMG 14545^T (MRS-9t)	nd
L. pseudomesenteroides 1145	nd	nd	*P. aeruginosa* FMCC B-26 (mye-9)
L. pseudomesenteroides 1249	*S. thermophilus* ACA-DC 4 (MRS-12)	*S. pneumoniae* LMG 14545^T (MRS-7t)	nd
E. faecium 1117	*S. thermophilus* ACA-DC 4 (M17-9)	nd	*P. aeruginosa* FMCC B-26 (M17-bl, mye-bl)
E. faecium 1138	*L. sakei* ACA-DC 2313 (MRS-18; mye-15);<br>*S. thermophilus* ACA-DC 4 (MRS-11)	nd	*L. innocua* LMG 11387^T (MRS-20, mye-15);<br>*L. innocua* LMG 13568 (MRS-20, mye-15);

			L. welshimeri 15008 (MRS-15, mye-12)
E. faecium 1154	*L. sakei* ACA-DC 2313 (MRS-18; mye-18); *S. thermophilus* ACA-DC 4 (MRS-15); *E. faecalis* LMG 11396 (MRS-9; mye-9)	nd	*L. innocua* LMG 11387^T (MRS-12, mye-12); *L. innocua* LMG 13568 (MRS-13, mye-13);*L. welshimeri* 15008 (MRS-11, mye-12)
E. faecalis 1168	*S. thermophilus* ACA-DC 4 (M17-15); *E. faecalis* LMG 11396 (M17-11)	nd	nd
E. faecalis 1169	*S. thermophilus* ACA-DC 4 (M17-15); *E. faecalis* LMG 11396 (M17-11)	nd	nd
E. faecalis 1172	*S. thermophilus* ACA-DC 4 (M17-15); *E. faecalis* LMG 11396 (M17-11)	nd	nd
E. faecium 1187	*L. sakei* ACA-DC 2313 (MRS-15, mye-18); *S. thermophilus* ACA-DC 4 (M17-15); *E. faecalis* LMG 11396 (mye-9)	nd	*L. innocua* LMG 11387^T (MRS-12, mye-11); *L. innocua* LMG 13568 (MRS-14, mye-11); *L. welshimeri* 15008 (MRS-10, mye-12)
E. faecalis 1188	*L. sakei* ACA-DC 2313 (MRS-15, mye-18); *S. thermophilus* ACA-DC 4 (M17-15); *E. faecalis* LMG 11396 (mye-11)	nd	*L. innocua* LMG 11387^T (MRS-13, mye-10); *L. innocua* LMG 13568 (MRS-14, mye-11); *L. welshimeri* 15008 (MRS-10, mye-12)
E. faecalis 1189	*L. sakei* ACA-DC 2313 (MRS-12, mye-14); *S. thermophilus* ACA-DC 4 (M17-15); *E. faecalis* LMG 11396 (M17-9, mye-11)	nd	*L. innocua* LMG 11387^T (MRS-12, mye-16); *L. innocua* LMG 13568 (MRS-13, mye-16); *L. welshimeri* 15008 (MRS-9, mye-12)
E. faecalis 1190	*S. thermophilus* ACA-DC 4 (M17-15); *E. faecalis* LMG 11396 (M17-11)	nd	

E. faecalis 1191	*S. thermophilus* ACA-DC 4 (M17-15);<br>*E. faecalis* LMG 11396 (M17-9)	nd	nd
E. faecium 1197	*S. thermophilus* ACA-DC 4 (M17-12)	*S. oralis* LMG 14532^T (MRS 10; mye-10t)	*L. welshimeri* 15008 (mye-7)
E. faecium 1198	*S. thermophilus* ACA-DC 4 (M17-12)	*S. oralis* LMG 14532^T (MRS-10t)	*L. welshimeri* 15008 (mye-9)
E. faecium 1200	*S. thermophilus* ACA-DC 4 (M17-12)	*S. oralis* LMG 14532^T (MRS-10; mye-10t)	*P. aeruginosa* FMCC B-26 (M17-bl)
E. faecium 1201	*S. thermophilus* ACA-DC 4 (M17-12)	nd	*P. aeruginosa* FMCC B-26 (mye-bl)
E. faecalis 1203	*L. sakei* ACA-DC 2313 (M17-17; mye-14);<br>*E. faecalis* LMG 11396 (M17-8, mye-8)	nd	*L. innocua* LMG 11387^T (MRS-18, mye-11);<br>*L. innocua* LMG 13568 (MRS-19, mye-12);<br>*L. welshimeri* 15008 (MRS-13, mye-9)
E. faecalis 1204	*S. thermophilus* ACA-DC 4 (M17-7)	nd	*P. aeruginosa* FMCC B-26 (mye-bl)
E. faecium 1214	*S. thermophilus* ACA-DC 4 (M17-12)	*S. oralis* LMG 14532^T (M17-9t)	nd
E. faecium 1216	*S. thermophilus* ACA-DC 4 (M17-12)	nd	*P. aeruginosa* FMCC B-26 (mye-bl)
E. faecium 1217	*S. thermophilus* ACA-DC 4 (M17-12)	*S. sanguinis* DSM 20068 (M17-9t);<br>*S. oralis* LMG 14532^T (M17-9t)	nd
E. faecium 1218	*L. sakei* ACA-DC 2313 (M17-17, mye-15);<br>*S. thermophilus* ACA-DC 44 (17-10);<br>*E. faecalis* LMG 11396 (M17-9, mye-9)	*S. sanguinis* DSM 20068 (M17-9t)	*L. innocua* LMG 11387^T (M17-15, mye-7);<br>*L. innocua* LMG 13568 (M17-15, mye-9);<br>*L. welshimeri* 15008 (M17-10, mye-8)
E. faecium 1219	*L. sakei* ACA-DC 2313 (M17-17, mye-15);<br>*S. thermophilus* ACA-DC 44 (17-10);<br>*E. faecalis* LMG 11396 (M17-9, mye-9)	nd	*L. innocua* LMG 11387^T (M18-15, mye-12);<br>*L. innocua* LMG 13568 (M17-15, mye-12);<br>*Li. welshimeri* 15008 (M17-12, mye-9)

E. faecium 1220	*L. sakei* ACA-DC 2313 (M17-15, mye-15);<br>*S. thermophilus* ACA-DC 4 (M17-10);<br>*E. faecalis* LMG 11396 (M17-9, mye-9)	nd	*L. innocua* LMG 11387^T (M17-16, mye-14)<br>*L. innocua* LMG 13568 (M17-16, mye-13);<br>*L. welshimeri* 15008 (M17-12, mye-12)
E. faecium 1221	*L. sakei* ACA-DC 2313 (M17-20, mye-15);<br>*S. thermophilus* ACA-DC 4 (M17-10);<br>*E. faecalis* LMG 11396 (M17-9, mye-9)	nd	*L. innocua* LMG 11387^T (M17-16, mye-12)<br>*L. innocua* LMG 13568 (M17-15, mye-12);<br>*L. welshimeri* 15008 (M17-12, mye-9)
E. faecium 1222	*L. sakei* ACA-DC 2313 (M17-15, mye-15);<br>*S. thermophilus* ACA-DC 4 (M17-10);<br>*E. faecalis* LMG 11396 (M17-9, mye-9)	nd	*L. innocua* LMG 11387^T (M17-14, mye-12);<br>*L. innocua* LMG 13568 (M17-14, mye-10);<br>*L. welshimeri* 15008 (M17-11, mye-9)
E. faecium 1233	*S. thermophilus* ACA-DC 4 (MRS-12)	*S. oralis* LMG 14532^T (M17-10t)	nd
E. faecium 1235	*S. thermophilus* ACA-DC 4 (MRS-12)	*S. oralis* LMG 14532^T (M17-10t)	nd
E. faecium 1268	*S. thermophilus* ACA-DC 4 (M17-12)	*S. pneumoniae* LMG 14545^T (M17-7)	nd
H. paralvei 1123	nd	*S. mutans* LMG 14558^T (MRS-9t);<br>*S. oralis* LMG 14532^T (MRS-9t)	*L. innocua* LMG 11387^T (MRS-14, mye-10);<br>*L. innocua* LMG 13568 (MRS-11, mye-9)
H. paralvei 1124	*L. sakei* ACA-DC 2313 (MRS-15);<br>*S. thermophilus* ACA-DC 4 (MRS-9)	nd	*L. innocua* LMG 11387^T (MRS 18, mye-8);<br>*L. innocua* LMG 13568 (MRS 18, mye-9);<br>*L. welshimeri* 15008 (MRS-7)
H. paralvei 1125	nd	nd	*L. innocua* LMG 11387^T (MRS 15, mye-10);<br>*L. innocua* LMG 13568 (MRS 12, mye-9)
H. paralvei 1126	*S. thermophilus* ACA-DC 4 (MRS-11)	*S. oralis* LMG 14532^T (MRS-8t)	

H. paralvei 1127	*L. sakei* ACA-DC 2313 (MRS-18, mye-14);<br>*S. thermophilus* (MRS-11)	*S. oralis* LMG 14532^T (MRS-8t);<br>*S. pneumoniae* LMG 14545^T (MRS-8t)	*L. innocua* LMG 11387^T (MRS 20, mye-15);<br>*L. innocua* LMG 13568 (MRS 20, mye-15);<br>*L. welshimeri* 15008 (MRS 14, mye-12)
Pseudomonas spp. 1146	nd	nd	*B. subtilis* FMCC B-109 (MRS-11);<br>*B. cereus* LMG 6923^T (mye-10t)
Pseudomonas spp. 1192	*L. lactis* LMG 6890^T (MRS-7, mye-9);<br>*S. thermophilus* ACA-DC 4 (MRS-11, mye-9);<br>*E. faecalis* LMG 11396 (MRS-9, mye-9)	*S. salivarius* LMG 11489^T (MRS-8, mye-10);<br>*S. sanguinis* DSM 20068 (MRS-13, mye-14);<br>*S. sobrinus* LMG 14641^T (MRS-10, mye-11);<br>*S. gordonii* LMG 14518^T (MRS-12, mye-13);<br>*S. mutans* LMG 14558^T (MRS-10, mye-11);<br>*S. oralis* LMG 14532^T (MRS-10, mye-12);<br>*S. agalactiae* LMG 14694^T (MRS-10, mye-12);<br>*S. anginosus* LMG 14502^T (MRS-9, mye-11);<br>*S. pneumoniae* LMG 14545^T (MRS-11, mye-12);<br>*S. pyogenes* LMG 21599^T (MRS-11, mye-13);	*L. welshimeri* 15008 (MRS-7, mye-9);<br>*B. subtilis* FMCC B-109 (mye-8);<br>*B. cereus* LMG 6923^T (mye-15)
Pseudomonas spp. 1195	*L. sakei* ACA-DC 2313 (mye-7);<br>*L. lactis* LMG 6890^T (MRS-7, mye-9);<br>*S. thermophilus* ACA-DC 4 (MRS-12, mye-10);<br>*E. faecalis* LMG 11396 (mye-8)	*S. salivarius* LMG 11489^T (MRS-10, my-12);<br>*S. sanguinis* DSM 20068 (MRS-10, mye-13);<br>*S. sobrinus* LMG 14641^T (MRS-10, mye-12);<br>*S. gordonii* LMG 14518^T (MRS-9, mye-13);<br>*S. mutans* LMG 14558^T (MRS-10, mye14);<br>*S. oralis* LMG 14532^T (MRS-14, mye-15);	*L. welshimeri* 15008 (MRS-9, mye-12)

		S. agalactiae LMG 14694^T (MRS-10, mye-14);<br>*S. anginosus* LMG 14502^T (MRS-8, mye-11);<br>*S. pneumoniae* LMG 14545^T (MRS-11, mye-13);<br>*S. pyogenes* LMG 21599^T (MRS-10, mye-13);	
Pseudomonas spp. 1212	*S. thermophilus* ACA-DC 4 (M17-12)	*S. sanguinis* DSM 20068 (MRS-10t);<br>*S. sobrinus* LMG 14641^T (MRS-8t);<br>*S. pneumoniae* LMG 14545^T (MRS-10)	nd
B. subtilis 1176	*L. lactis* LMG 6890^T (mye-12t);<br>*S. thermophilus* ACA-DC 4 (MRS-15)	*S. agalactiae* LMG 14694^T (MRS-8);<br>*S. pneumoniae* LMG 14545^T (MRS-10t)	nd
B. subtilis 1225	*S. thermophilus* ACA-DC 4 (MRS-12)	*S. pneumoniae* LMG 14545^T (MRS-10)	nd
B. subtilis 1241	*L. sakei* ACA-DC 2313 (MRS-14);<br>*S. thermophilus* ACA-DC 4 (MRS-12)	*S. oralis* LMG 14532^T (MRS-7t)	*L. welshimeri* 15008 (MRS-8)
B. subtilis 1242	*L. sakei* ACA-DC 2313 (MRS-12);<br>*S. thermophilus* ACA-DC 4 (MRS-12)	*S. oralis* LMG 14532^T (MRS 8);<br>*S. pneumoniae* LMG 14545^T (MRS-11)	nd
B. subtilis 1243	*S. thermophilus* ACA-DC 4 (MRS-12)	*S. pneumoniae* LMG 14545^T (MRS-9)	nd
B. subtilis 1244	*L. sakei* ACA-DC 2313 (MRS-17);<br>*S. thermophilus* ACA-DC 4 (MRS-12)	*S. oralis* LMG 14532^T (MRS-8)	nd

bl: border line inhibition, t: turbid inhibition zone, mye: milk supplemented with yeast extract, nd: not detected.
